# Composition, Electronic and Magnetic Investigation of the Encapsulated ZnFe_2_O_4_ Nanoparticles in Multiwall Carbon Nanotubes Containing Ni Residuals

**DOI:** 10.1186/s11671-015-0971-7

**Published:** 2015-06-11

**Authors:** Saja Al Khabouri, Salim Al Harthi, Toru Maekawa, Yutaka Nagaoka, Mohamed E Elzain, Ashraf Al Hinai, AD Al-Rawas, AM Gismelseed, Ali A Yousif

**Affiliations:** Department of Physics, Sultan Qaboos University, Muscat, PC 123 Sultanate of Oman; Bio-Nano Electronics Research Center, Toyo University, 2100, Kujirai, Kawagoe, Saitama 350 8585 Japan; Department of Chemistry, Sultan Qaboos University, Muscat, PC 123 Sultanate of Oman

**Keywords:** Surface, Carbon nanotubes, Metallic, Encapsulation, Charge transfer, Ferromagnetism, Distinct blocking temperatures

## Abstract

We report investigation on properties of multiwall carbon nanotubes (mCNTs) containing Ni residuals before and after encapsulation of zinc ferrite nanoparticles. The pristine tubes exhibit metallic character with a 0.3 eV reduction in the work function along with ferromagnetic behavior which is attributed to the Ni residuals incorporated during the preparation of tubes. Upon encapsulation of zinc ferrite nanoparticles, 0.5 eV shift in Fermi level position and a reduction in both the π band density of state along with a change in the hybridized sp^2^/sp^3^ ratio of the tubes from 2.04 to 1.39 are observed. As a result of the encapsulation, enhancement in the σ bands density of state and coating of the zinc ferrite nanoparticles by the internal layers of the CNTs in the direction along the tube axis is observed. Furthermore, Ni impurities inside the tubes are attracted to the encapsulated zinc ferrite nanoparticles, suggesting the possibility of using these particles as purifying agents for CNTs upon being synthesized using magnetic catalyst particles. Charge transfer from Ni/mCNTs to the ZnFe_2_O_4_ nanoparticles is evident via reduction of the density of states near the Fermi level and a 0.3 eV shift in the binding energy of C 1 s core level ionization. Furthermore, it is demonstrated that encapsulated zinc ferrite nanoparticles in mCNTs resulted in two interacting sub-systems featured by distinct blocking temperatures and enhanced magnetic properties; i.e., large coercivity of 501 Oe and saturation magnetization of 2.5 emu/g at 4 K.

## Background

Since the discovery of carbon nanotubes (CNTs), there has been great interest in the synthesis and characterization of CNTs composites [1]. For instance, encapsulation of magnetic materials in CNTs offers great potential since for particle applications carbon shielding of magnetic materials provides a stable coating against oxidation and degradation. Although the intrinsic properties of the CNTs such as nanometric cross section, high aspect ratio, good thermal, and electrical conductivity suggest high application potential [2], the magnetic properties of the composites are effected by residual magnetic impurities originating from the catalyst used to prepare the CNTs. Depending on the catalyst used to prepare the CNTs, the catalyst particles are left as residuals in the tubes and accordingly the magnetic properties of the CNTs are influenced [3].

Zinc ferrite is a promising microwave absorber; however, it is quite heavy due to high density, which restricts its utilization in applications requiring lightweight materials [4]. ZnFe_2_O_4_/CNTs composites may provide immediate advantage over ZnFe_2_O_4_ nanoparticles because of the relatively low density of the composites. In addition, it has been demonstrated that encapsulation of paramagnetic particles into CNTs leads to paramagnetic needles whose movements can be controlled [5]. Despite these advantages, encapsulating ferrites inside the nanotubes lead to effects which are not yet fully understood and explored. For example, in addition to ambiguous electronic and magnetic effects of the catalyst used to prepare the CNTs, it is unclear if there will be electron transfer from the host CNTs to the encapsulated ferrites or vice versa. Also, it is anticipated that stress in CNTs with small inner diameters (IDs) causes deformation of the encapsulated particles, consequently affecting their magnetic properties. In addition to that, the nanoparticles can adopt the internal shape of the CNTs and accommodate themselves along the tubes axis leading to enhancement of the magnetic anisotropy, which affects the blocking temperature of the composite system. Furthermore, the tendency of nanoparticles to agglomerate inside the tubes can influence the magnetic properties rendering them similar properties of large particles.

Wet chemistry has been used to grow metal oxides such as NiO and Nd_2_O_3_ inside the inner cavity of the CNTs [6]. This study is the first to use the aforementioned method successfully to synthesise zinc ferrite multiwall CNTs composites in order to investigate their structural, compositional, electronic, and magnetic properties. The use of multiwall CNTs (mCNTs) containing residual Ni impurities reflects the reality that catalysts are needed for the production of most of the CNTs. Details of electron transfer, quality of CNTs, oxidation states of zinc and iron inside the composite, and magnetic properties at various temperatures are discussed.

## Methods

### Synthesis

The mCNTs with ID = 5–10 nm and outer diameter (OD) = 20–30 nm were purchased from Chinese Academy of Sciences (CAS) synthesized via chemical vapor deposition (CVD) in the presence of nickel catalyst. A round bottom flask containing a sample of CNTs (0.5 g), Zn(NO_3_)_2_ 6H_2_O (98 %, BDH, 2.0 g), and Fe(NO_3_)_3_ 9H_2_O (98 %, BDH, 5.4 g) dissolved in azeotropic nitric acid (68 %, ca. 30.0 ml) was heated to reflux for 4.5–5 h. The molar ratio of Zn(NO_3_)_2_ to Fe(NO_3_)_3_ was 1:2. The nitric acid solution was decanted off, and the black sludge was pipetted onto glass filter paper. The sample was dried overnight in an oven at 60 °C and then calcined by heating in a stream of N_2_ at 400 °C for 4 h for the conversion of the nitrate to the corresponding zinc ferrite according to the following equation:

$$ 2\mathrm{F}\mathrm{e}{\left(\mathrm{N}{\mathrm{O}}_3\right)}_3\kern0.5em +\kern0.5em \mathrm{Z}\mathrm{n}{\left(\mathrm{N}{\mathrm{O}}_3\right)}_2\kern0.5em \overset{400{}^{\circ}C}{\to}\kern0.5em \mathrm{Z}\mathrm{n}\mathrm{F}{\mathrm{e}}_2{\mathrm{O}}_4\kern0.5em +\kern0.5em 8\mathrm{N}{\mathrm{O}}_2\kern0.5em +\kern0.5em 2{\mathrm{O}}_2 $$

### Characterization

X-ray diffraction (XRD) measurements were carried out in a Philips PW 1700 diffractometer with CuKα source (λ = 0.154060 nm). The high resolution transmission electron microscopy (HR-TEM) and energy dispersive x-ray spectroscopy (EDS) elemental mapping were performed on a (JEOL JEM-ARM200F) instrument. The images were acquired at bright field and dark field scanning transmission electron microscopy (STEM) at 80 kV capabilities. The magnetization was measured with a DMS 1660 vibrating sample magnetometer (VSM) in a magnetic field up to 13 kOe. Superconducting Quantum Interference Device (SQUID) (Quantum Design) is used to measure the magnetic properties at 77 and 4 K and field and zero field cooling curves. Mossbauer spectra were recorded on the powder sample using a constant-acceleration spectrometer with 50 mCi 57Co in Rh source. The X-ray photoelectron spectroscopy (XPS) and ultraviolet photoemission (UPS) measurements were carried out using an Omicron Nanotechnology system (Omicron Nanotechnology Gmbhm Taunusstein, Germany). The XPS radiation was a monochromatic Al Kα radiation of hν = 1486.6 eV [7]. The chemical composition was extracted from the wide scan using CASA XPS software (Fairly, N. CASA XPS, version 2.0; CASA Software Ltd., Devon, UK). The fitting of the spectrum was done by Gaussian-Lorentzian functions with a Shirley background subtraction. In order to avoid charging effects during the XPS scans, electron gun flooding was used for charge compensation. A He lamp with 21.2 eV (He Ι) excitation energy was used for the UPS analysis. Indium tin oxide (ITO) was used as a standard sample to check the validity of the work function value estimated following the procedure reported in ref [8].

## Results and Discussion

### Multi Wall Carbon Nanotubes

**Fig. 1:**
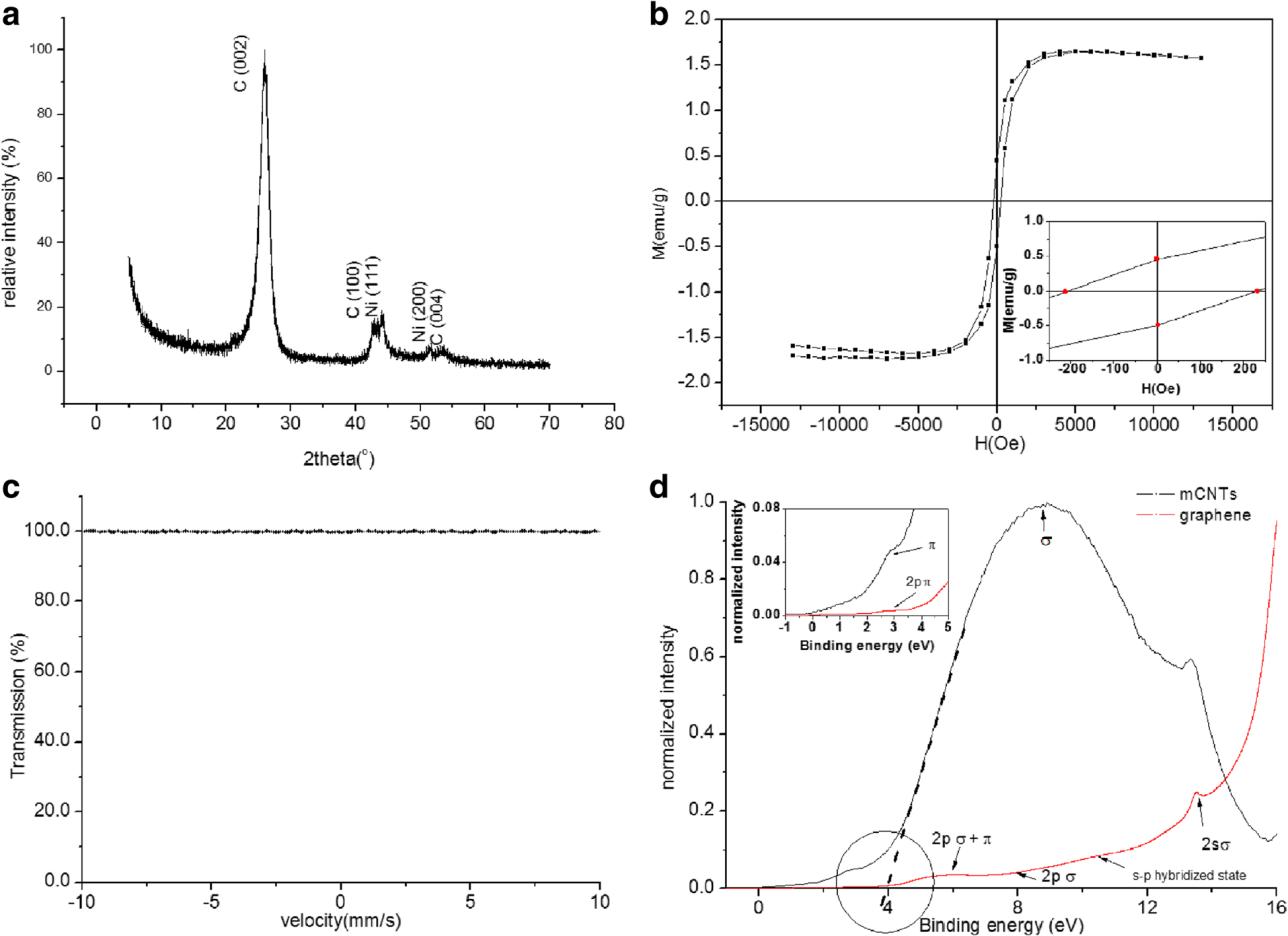
**a** XRD pattern of the multi wall carbon nanotubes (mCNTs). **b** Magnetization curve up to 13 kOe at room temperature of the (mCNTs). Inset is enlargement of the hysteresis loop at low applied fields. **c** Mossbauer spectrum for the mCNTs recorded at room temperature. **d** UPS valence band spectra of the mCNTs and few layers of graphene. Inset is the density of state (DOS) at vicinity of Fermi level

The XRD pattern of the mCNTs is presented in Fig. 1a. The XRD pattern reveals the presence of three peaks corresponding to (002), (100), and (004) plane reflections of the carbon atoms in good agreement with the earlier reports [9]. The crystal plane diffraction peaks of Ni (111) and (200) are detected indicating the presence of Ni impurities in the mCNTs. The diffraction peaks of Ni are smaller than the diffraction peaks of carbon in the mCNTs indicating smaller volume fractions of Ni impurities. Figure 1b shows the magnetization curve (up to 13 kOe) of the mCNTs at room temperature. The value of saturation magnetization allows to estimate the amount of the magnetic material in the sample [3]. Therefore, the mCNTs show weak magnetic effect, with a magnetization of 1.6 emu/g at 13 kOe due to small traces of the Ni magnetic impurities similar to other observations reported in literature [10]. As depicted from the inset of Fig. 1b, the remanence of 5 mg mCNTs is found to be 0.474 emu/g and the coercivity is 218 Oe. Lipert et al. [3] reported ferromagnetic properties of different mCNTs where the magnetic behavior was explained by the encapsulation of the catalyst particles such as Co and FeCo in the nanotubes. The values of coercivity found by Lipert are consistent with the large values of coercivity found for the mCNTs used in this study. Lipert assumed that the catalyst particles encapsulated in the CNTs exhibit single domain states in order to explain the large values of coercivity and found that the coercivity depends on the magneto-crystalline anisotropy constant of the catalyst particles. The ^57^Fe Mössbauer spectrum for the mCNTs is shown in Fig. 1c.

No absorption of gamma rays is detected in the spectrum indicating the absence of Fe content in the mCNTs.

Figure 1d presents the valence band data obtained by UPS for mCNTs and for a sample of few layers of graphene acquired by peeling layers from highly oriented pyrolytic graphite (HOPG ZYA) (i.e., the few layers of graphene data are included for comparison and clarity in explanations). The spectrum of the graphene reveals five C 2p and C 2 s band features associated with the crystalline state of the material: (1) 2p π at ~2.6 eV, (2) crossing of 2p π and 2p σ bands at 5.9 eV, (3) 2p σ broad band feature around 7.9 eV, (4) 2 s-2p hybridized state at 10.4 eV, and (5) 2 s σ band at 13.4 eV [11]. The observed π band feature for the mCNTs is small, and the σ band appears broad. The CNT UPS spectrum can be understood as angle integrated spectra of graphene. Since the nanotube is a rolled graphene sheet, photoelectrons ejected from both normal and tangential directions of the nanotube/graphene surface are simultaneously detected [12]. The work function was determined by the intersection of the high binding energy cut-off of the accelerated electrons with the base line of the spectrum (as shown in Fig. 1d and labeled by a circle. The work function of the mCNTs used in this study was found to be (4.0 ± 0.1 eV) suggesting metallic character of CNTs. This value is 0.3 eV lower than that of the reported value of 4.3 eV for the purified mCNTs and attributed to the effect of Ni impurities in the mCNTs. Similar reduction in the work function has been reported by Giusca et al. [13] upon filling CNTs with GeTe. Theoretically [14], the work function will reduce due to charge transfer from metal to CNT, which shifts the Fermi level of conduction band towards the vacuum. Experimental realization of this shift and charge transfer is revealed in the inset of Fig. 1d. The charge transfer is reflected in the increase of the density of state intensity near the Fermi level (i.e., from 0 to 3 eV) of the mCNTs in comparison to that of graphene layers and provides a strong indication of the metallic character of the tubes.

**Fig. 2:**
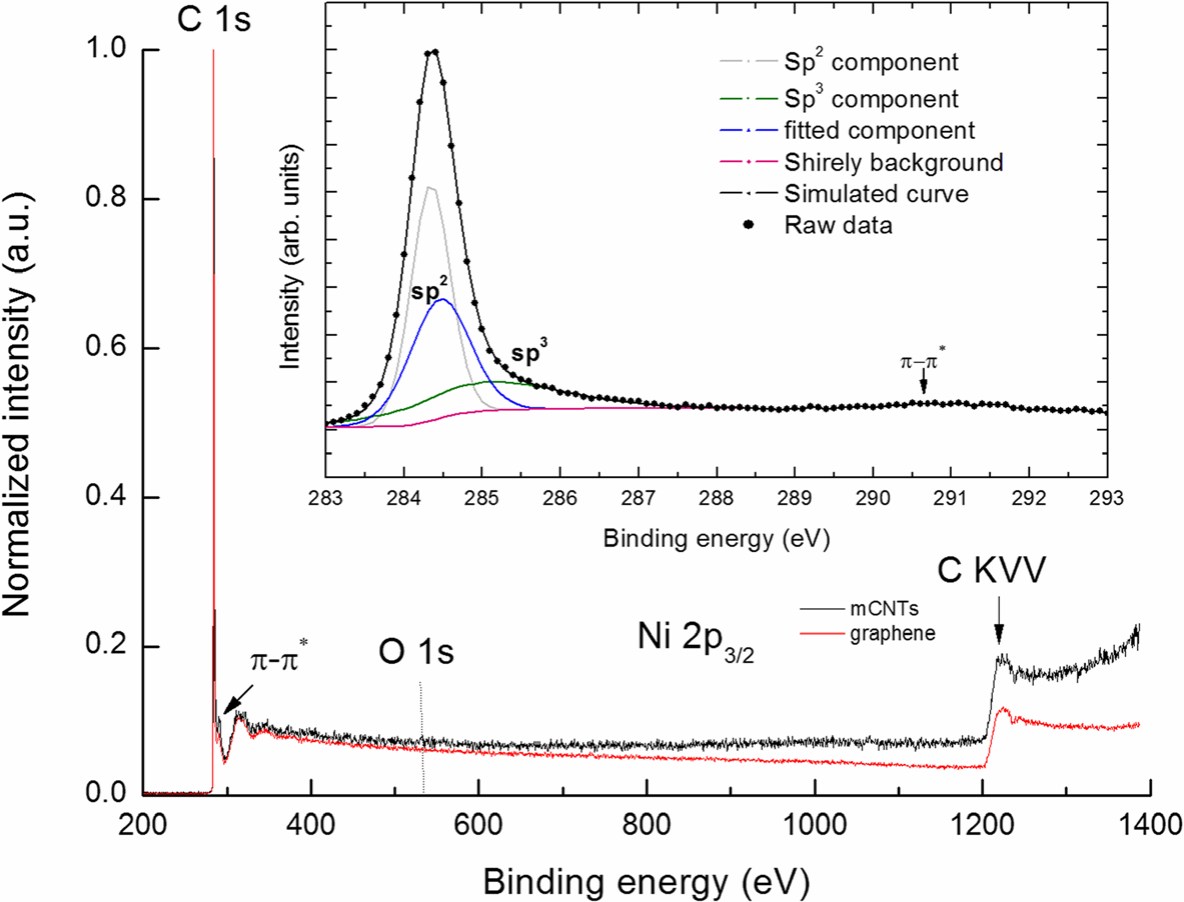
The overall XPS spectra of mCNTs and few layers of graphene. Deconvolution of the XPS spectrum of the C1s carbon level of the mCNTs (inset)

Figure 2 shows the XPS survey spectra recorded for mCNTs and graphene. The commonly observed π-π^*^ transition is positioned in the mCNTs at 290.5 eV for both samples. Oxygen and nickel are not detected in the mCNTs. This could be due to small oxygen and Ni concentrations below XPS detection limit (i.e., 0.1 %). C 1 s core level excitations of mCNTs are shown as an inset of Fig. 2. The simulation using CASA XPS Software suggests peaks positioned at 284.3 and 285.1 eV corresponding to sp^2^ and sp^3^ hybridized states, respectively. Quantification of sp^2^/sp^3^ ratio reveals the dominance of sp^2^ hybridization (i.e., sp^2^/sp^3^ = 2.04, where sp^2^ = 67 % and sp^3^ = 33 %), which is characteristic of a two-dimensional sheet of sp^2^ bonded carbon atoms [15]. The peak at 284.5 eV could be attributed to some carbon impurities left behind after sample preparation [16]. The D parameter gives an indication of the relative amounts of sp^2^ and sp^3^ carbon. The D parameter is defined as the separation in energy between the most positive and most negative excursions obtained from the first derivative Auger transition C KVV spectrum. In the spectrum of the mCNTs sample, the value of this parameter is 19.1 eV which agrees well with the value obtained for mCNTs obtained by Bolotov et al. [17]. The presence of sp^3^ carbon atoms may be associated with the defects caused by the presence of Ni impurities or the presence of a layer of turbostratic carbon [18, 19].

### ZnFe_2_O_4_/mCNTs

**Fig. 3:**
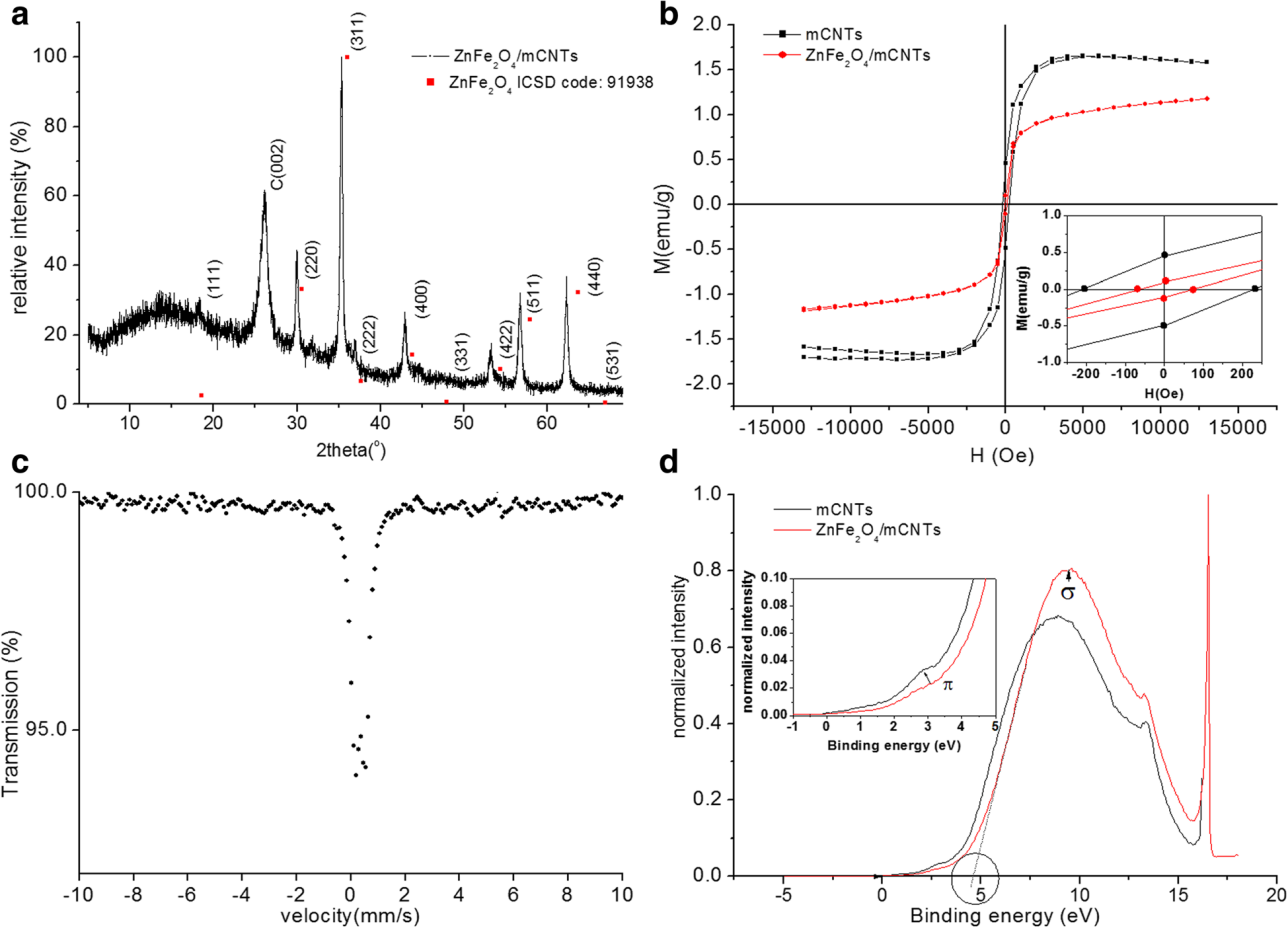
**a** XRD pattern of ZnFe_2_O_4_/mCNTs. **b** Magnetization curve up to 13 kOe at room temperature of ZnFe_2_O_4_/mCNTs. Inset is enlargement of the hysteresis loop at low applied fields. **c** Mossbauer spectrum for ZnFe_2_O_4_/mCNTs recorded at room temperature. **d** UPS valence band spectra of ZnFe_2_O_4_/mCNTs and mCNTs. Inset is the density of state DOS at vicinity of Fermi level

Having discussed the results obtained from mCNTs and effect of Ni residuals, we present the results obtained from the encapsulation of ZnFe_2_O_4_ in these tubes. The XRD peaks of ZnFe_2_O_4_/mCNTs correspond with the reported values of ZnFe_2_O_4_ (ICSD code; 91938) and carbon (002), see Fig. 3a. The XRD pattern of ZnFe_2_O_4_/mCNTs confirmed the cubic spinel structure of zinc ferrite; peaks are indexed as per ISCD pattern. The XRD pattern was analyzed by refining the experimental data using a standard Rietveld refinement technique. The lattice constant derived is 8.454 Ȧ, which is significantly larger than that of the ICSD reported value of a = 8.263 Ȧ. The estimated crystallite size from (311) peak is 13 nm.

Figure 3b shows the magnetization curve at room temperature for mCNTs and ZnFe_2_O_4_/mCNTs. The magnetization loop shows larger hysteresis for mCNTs than ZnFe_2_O_4_/mCNTs. The saturation magnetization of ZnFe_2_O_4_/mCNTs is 1.17 emu/g lower than that of the mCNTs. As shown in the inset of Fig. 3b, ZnFe_2_O_4_/mCNTs is found have a remanence of 0.104 emu/g and coercivity 70 Oe. The magnetization curve of ZnFe_2_O_4_ nanoparticles prepared by co-precipitation method shows super-paramagnetic behavior [20]. CNTs decorated with ZnFe_2_O_4_ nanoparticles [21] show an incremental value of magnetization compared to pure ZnFe_2_O_4_ nanoparticles. Therefore, coercivity shown by ZnFe_2_O_4_/mCNTs can be attributed to Ni impurities. The decrease in magnetic parameters of ZnFe_2_O_4_/mCNTs compared to mCNTs could be because less content of Ni impurities is present in ZnFe_2_O_4_/mCNTs sample than the amount in the same mass of mCNTs.

The ^57^Fe Mössbauer spectrum recorded for ZnFe_2_O_4_/mCNTs is shown in Fig. 3c. The main characteristic of the spectrum is the presence of a central paramagnetic doublet with isomer shift (δ) and quadruple splitting (ΔE_Q_) of (0.34 ± 0.02 mm/s) and (0.37 ± 0.02 mm/s), respectively, and (0.35 ± 0.02 mm/s) line width (Γ). The value of δ suggests the presence of only Fe^3+^ [22]. The δ, ΔE_Q_, and Γ values of bulk spinal zinc ferrite are 0.350, 0.333, and 0.258 mm/s [23], respectively. The line width of ZnFe_2_O_4_/mCNTs is higher than that of the bulk and that could be due to a distribution of the isomer shift and/or the quadrupole splitting as a consequence of random strain and surface effects and/or random size distribution. No change is observed in Mossbauer parameters when measurement was conducted at liquid nitrogen temperature (not shown).

Lattice strain could result in non-cubic symmetry and increase of the quadrupole splitting [24]. Figure 3d shows the UPS spectra of ZnFe_2_O_4_/mCNTs and mCNTs. Energy shift of the π and σ bands of ZnFe_2_O_4_/mCNTs with respect to mCNTs is observed. The DOS near the Fermi level of ZnFe_2_O_4_/mCNTs is slightly lower than that for mCNTs (see inset), indicating charge transfer between the Ni/mCNTs and the ZnFe_2_O_4_. Consequently, the work function of ZnFe_2_O_4_/mCNTs increased up to 4.6 eV which might lead to the increase of the electrical resistance of ZnFe_2_O_4_/mCNTs composite.

**Fig. 4:**
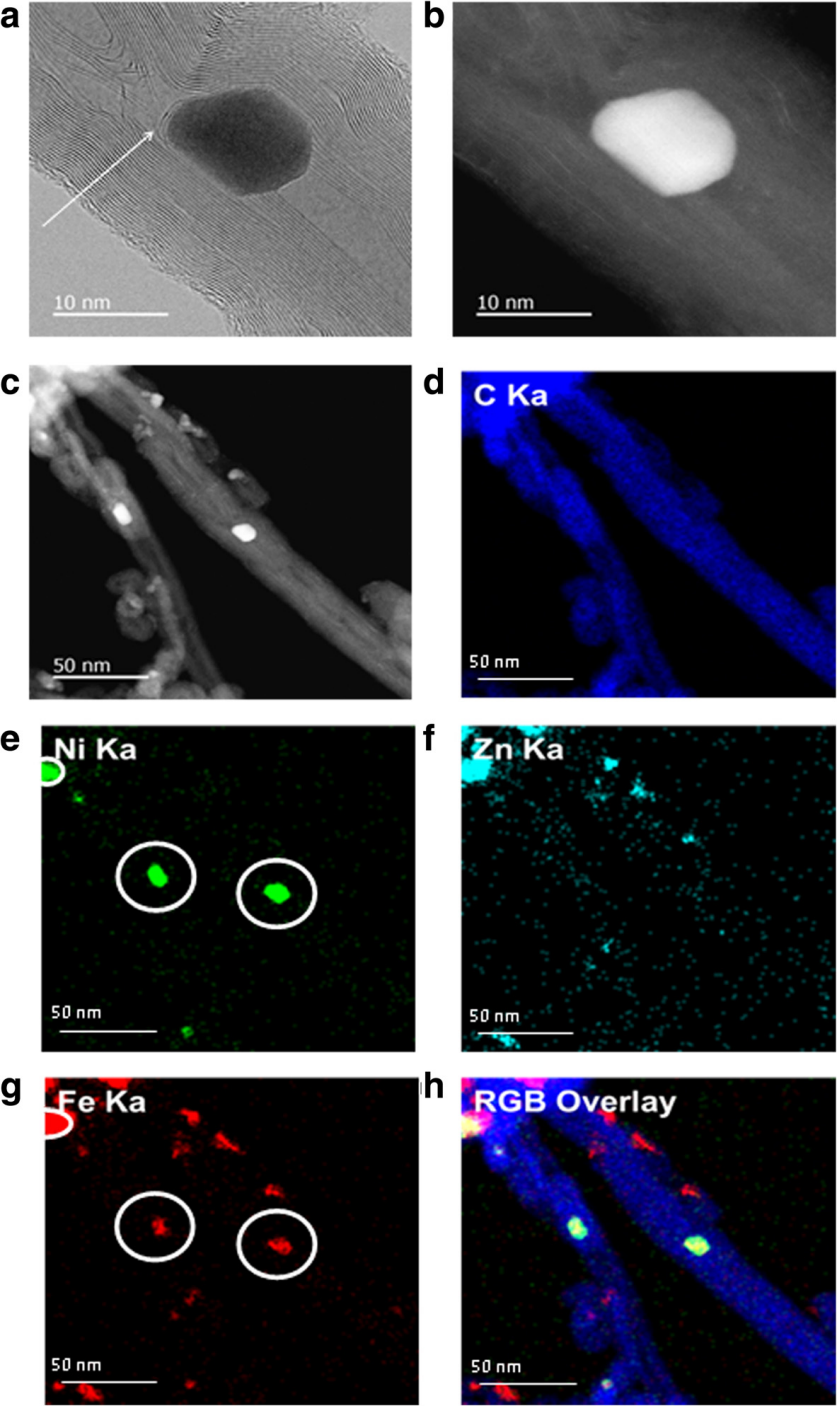
**a** STEM bright field image of single ZnFe_2_O_4_/mCNT. *Arrow* indicates the internal walls of mCNT partly coating ZnFe_2_O_4_ particle. **b** STEM dark field image of single ZnFe_2_O_4_/mCNT. **c** STEM annular dark-field image of ZnFe_2_O_4_/mCNTs. **d** The EDS x-ray maps of **e** nickel, the *circles* indicate Ni is agglomerated in the presence of ZnFe_2_O_4_. **f** Zinc. **g** Iron, the *circles* indicate the presence of iron in the same locations of Ni. **h** Display of the three specified maps as *red*, *green*, and *blue* overlays—representing iron, nickel, and carbon phase distributions

The STEM image shown in Fig. 4a reveals that the nanoparticles were encapsulated in the mCNTs. These particles are coated partly along the axis of the tube by its internal layers as seen in Fig. 4b. The particles attached to the tubes (Fig. 4c) are zinc ferrite crystals exterior to the tube cavities. Those are formed from the traces of the nitric acid left on the filter paper after filtration process [25]. Figure 4d–h shows the two-dimensional EDS elemental mapping images of ZnFe_2_O_4_/mCNTs. It can be further confirmed that ZnFe_2_O_4_ nanoparticles are present inside the inner cavity of mCNTs. Figure 4e shows that nickel is formed at ZnFe_2_O_4_ positions (Fig. 4f and g) which confirms the attraction of Ni by ZnFe_2_O_4_ nanoparticles and suggests possible mechanism of purification of CNTs synthesized using magnetic catalyst, e.g., Ni, Co, etc.

**Fig. 5:**
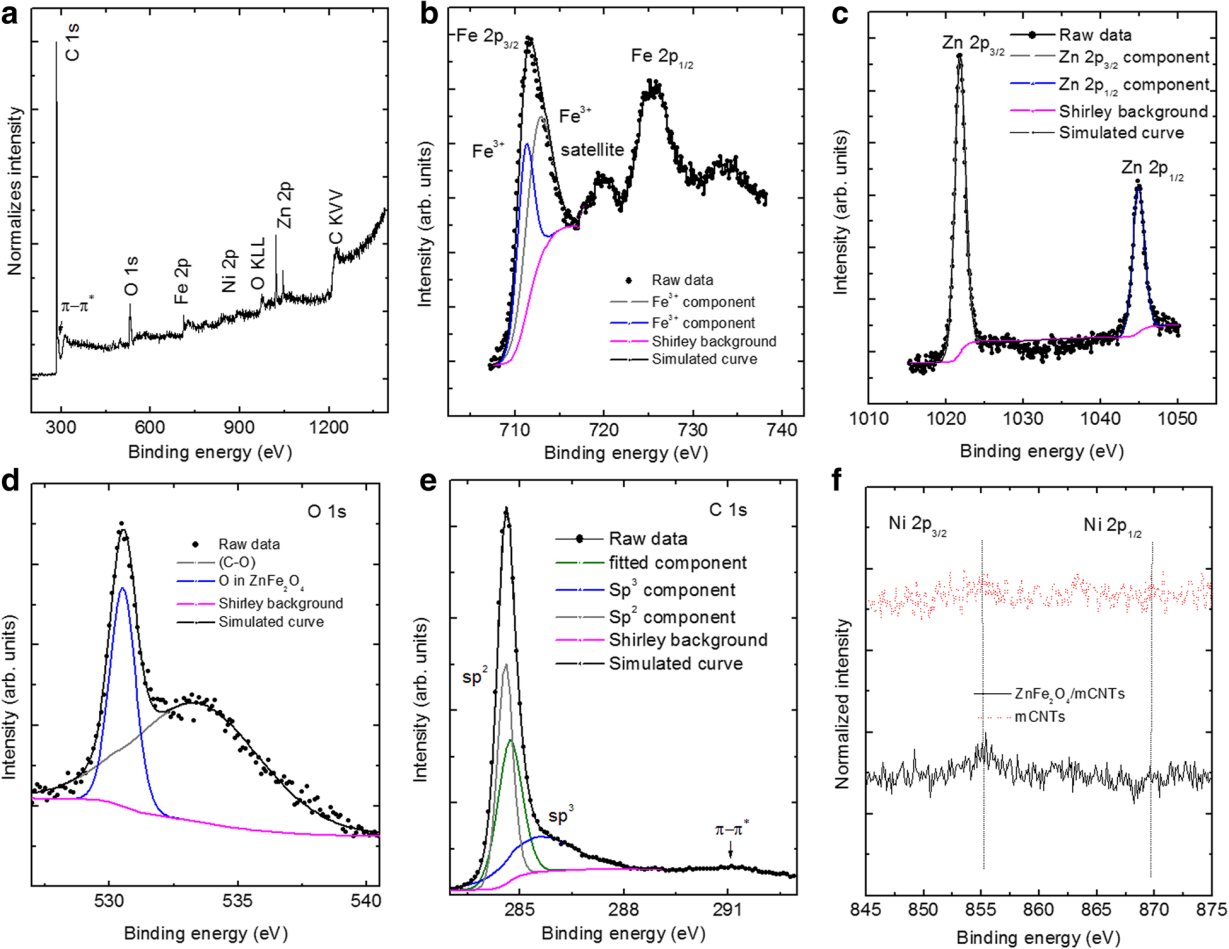
**a** XPS survey scan of the ZnFe_2_O_4_/mCNTs composite. *Narrow*-scan XPS spectra of the constituent elements in ZnFe_2_O_4_/mCNTs: **b** Fe 2p core-level, **c** Zn 2p core-level and **d** O1s core-level, **e** C1s core level, **f** Ni 2p core level spectra of ZnFe_2_O_4_/mCNTs and mCNTs as prepared

The wide scan XPS spectrum for ZnFe_2_O_4_/mCNTs is shown in Fig. 5a. The spectrum exhibits characteristic photoelectron lines of C, O, Zn, Fe, and Ni. This result confirms the formation of zinc ferrite ZnFe_2_O_4_. Figure 5b displays the Fe 2p photoelectron spectrum at binding energy of 711.4 eV. The 2p_3/2_ peak is deconvoluted into two peaks at binding energies of 711.1 and 712.6 eV illustrating the multiple characteristic of Fe^3+^ oxidation state. This is supported by the broad shape of the satellite peak observed at approximately 719 eV. Energy separation between the 2p_3/2_ and 2p_1/2_ was observed to be ~13 eV which generally associated with Fe^3+^ binding state [26, 27] and is in agreement with Mössbauer results. It should be mentioned that no evidence of oxidation reduction from Fe^3+^ to Fe^2+^ was observed as a result of ZnFe_2_O_4_/mCNTs sample preparation. The expected binding energy of 2p_3/2_ line of Fe^2+^ is at 709.5 eV [26] and well below our multiple Fe^3+^ oxidation state binding energy values of 711.1 and 712.6 eV.

Figure 5c shows a Zn 2p narrow scan XPS spectrum. The binding energies of Zn 2p_3/2_ and Zn 2p_1/2_ were 1021.8 and 1044.95 eV, respectively. These binding energies agree with the reported values of the binding energies of Zn^2+^ [27, 28]. The photoelectron peak positions and the sharp peaks indicate tetrahedral coordination of zinc in the ZnFe_2_O_4_ [28]. Asymmetric O 1 s spectrum is observed in Fig. 5d. Simulation results show that the spectrum is consisted of two components centered at 530.5 and 533.4 eV. The first is attributed to the oxygen in the ZnFe_2_O_4_ [26]. The peak at higher binding energy 533.4 eV is assigned to (C–O) oxygen singly bonded to carbon groups [29]. Deconvolution of C1s peak of ZnFe_2_O_4_/mCNTs is shown in Fig. 5e. As a result of preparing ZnFe_2_O_4_ inside the mCNTs, the proportion of sp^3^ hybridization of the carbon atoms increases by 9 % with respect to that in mCNTs alone and therefore the value of sp^2^/sp^3^ ratio decreases to 1.39. This change is associated with the damage observed from the STEM images in the internal walls of the mCNTs as a consequence of the growth of zinc ferrite particles inside the internal cavity of the mCNTs. It should be noted that there is a shift in the binding energy of C1s core level by 0.3 eV due to the presence of ZnFe_2_O_4_ nanoparticles indicating the existence of charge transfer between the ZnFe_2_O_4_ and the host mCNTs. The presence of Ni impurities in the ZnFe_2_O_4_/mCNTs is confirmed by the small Ni 2p_3/2_ and Ni 2p_1/2_ peaks observed in the narrow scan spectrum presented in Fig. 5f. After 100 short scans, Ni was detected in ZnFe_2_O_4_/mCNTs, which was not the case when the narrow scan was done for mCNTs alone which suggests that there is a tendency of agglomeration of Ni with the presence of ZnFe_2_O_4_.

**Fig. 6:**
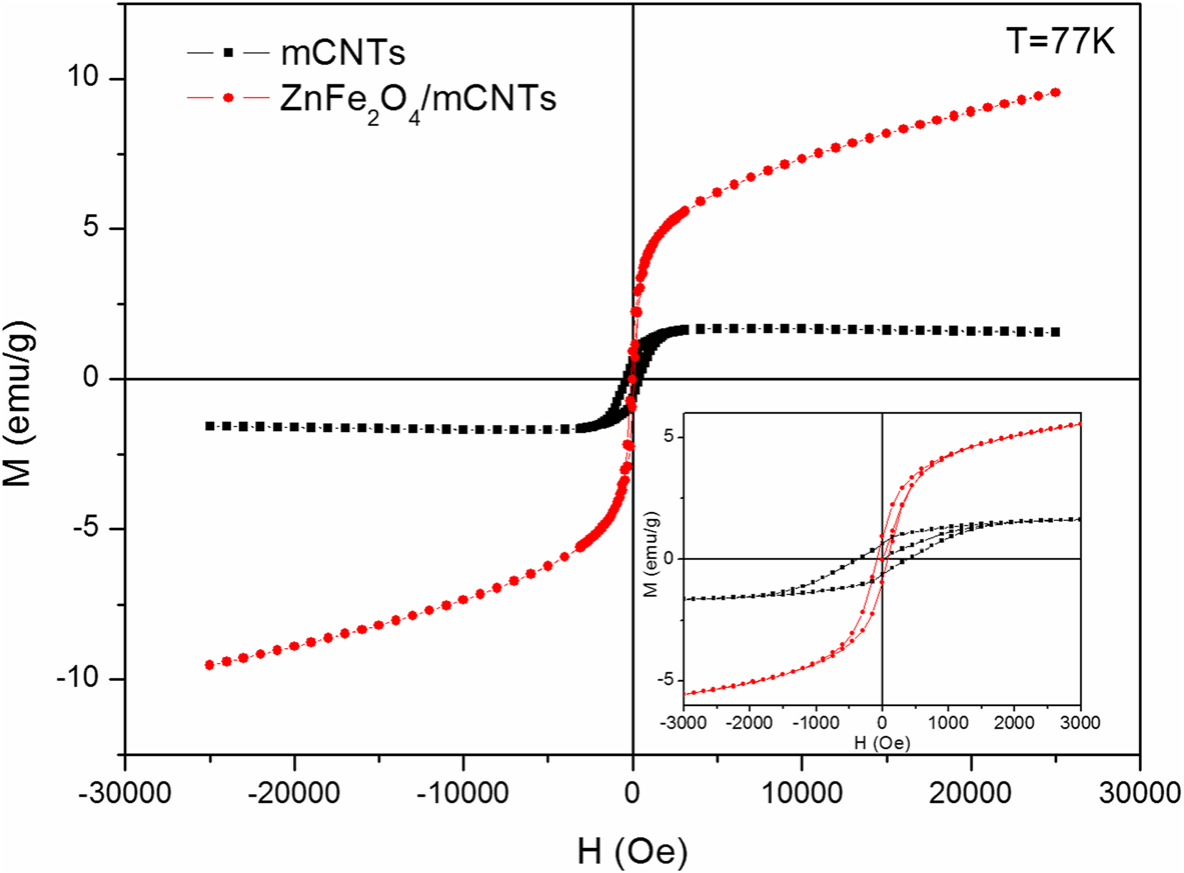
M-H loops of mCNTs and ZnFe_2_O_4_/mCNTs at 77 K. The inset shows an enlarged view of the M-H at 77 K

The M-H loops of mCNTs and ZnFe_2_O_4_/mCNTs measured at 77 K are presented in Fig. 6. The ZnFe_2_O_4_/mCNTs show weak ferromagnetism coming from the mCNTs dominated by paramagnetic behavior of the ZnFe_2_O_4_ nanoparticles. The coercivity of mCNTs alone at 77 K is around 380 Oe, and due to the paramagnetic dominant nature of ZnFe_2_O_4_ inside the composite, the coercivity of the composite decreases to 83.1 Oe.

**Fig. 7:**
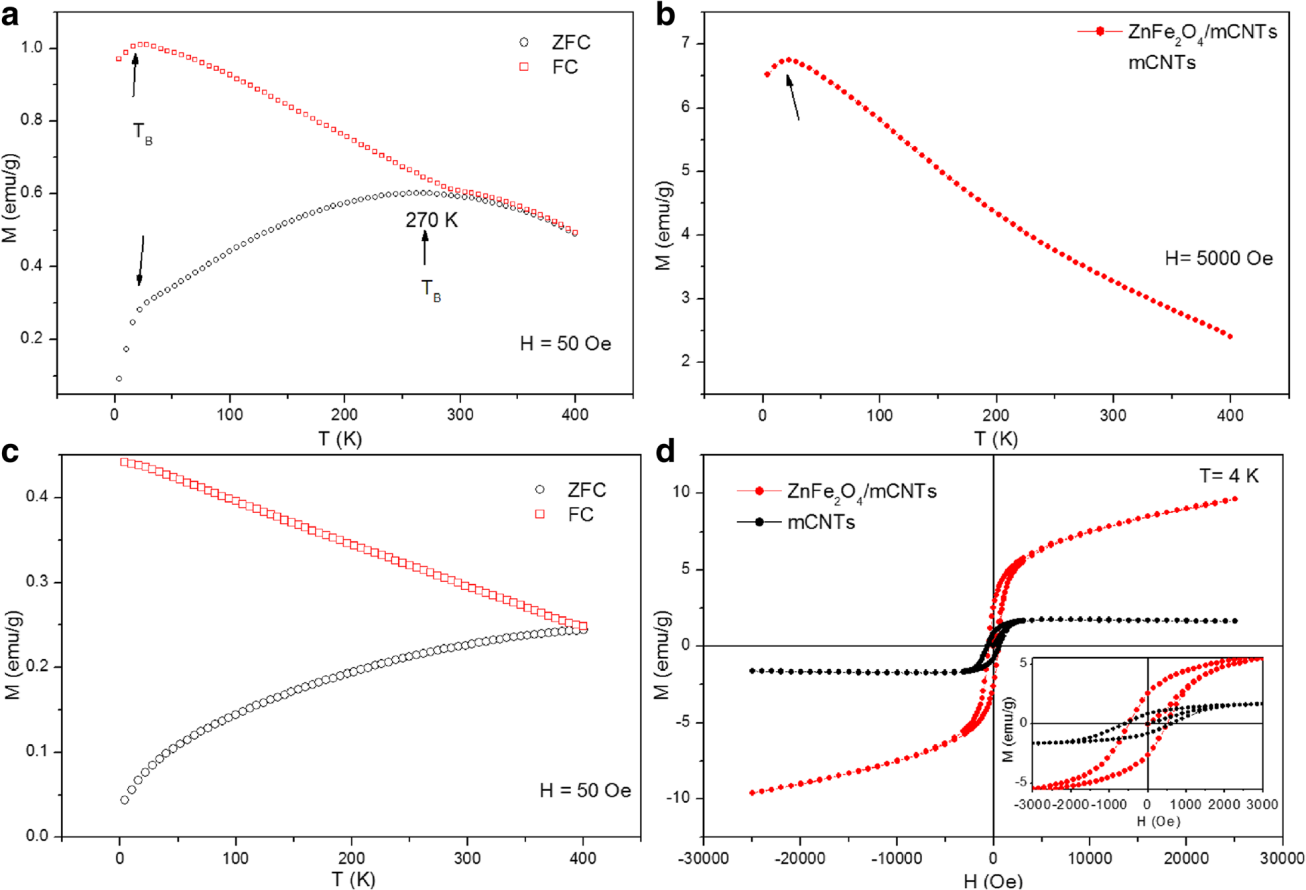
Magnetization measurements under ZFC and FC conditions in an applied magnetic field of 50 Oe for **a** ZnFe2O4/mCNTs and **c** mCNTs. **b** Magnetization versus temperature measurement of ZnFe2O4/mCNTs at an applied field of 5000 Oe. **d** M-H loop of ZnFe2O4/mCNTs at 4 K. The inset shows an enlarged view of the M-H at 4 K

Interestingly, the ZFC/FC magnetization versus temperature measurements in Fig. 7a displays two blocking temperatures of the ZnFe_2_O_4_/mCNTs composite, reflecting the behavior of two magnetic sub-systems, i.e., ZnFe_2_O_4_ nanoparticles and mCNTs with Ni impurities (mCNTs/Ni). The lower T_B_ at ~22 K is the blocking temperature of ZnFe_2_O_4_ nanoparticles—indicated by arrows in Fig. 7a, expressing the known behavior of the zinc ferrite nanoparticles of ~10 nm size [30, 31]. To verify the above observation, magnetization versus temperature measurement was acquired at a higher applied field, 5000 Oe, see Fig. 7b, where a sharp peak at 22 K is found. In addition, the discrepancy between the ZFC and FC at low magnetic field demonstrates the super-paramagnetic behavior of the ZnFe_2_O_4_ nanoparticles [32]. The higher observed T_B_ at 270 K gives experimental evidence of the existence of another magnetic sub-system characteristic of Ni residuals in mCNTs. The ZFC trend is similar to that of the reported NiFe_2_O_4_ nanoparticles of 40 nm size with T_B_ = 250 K [30]. Although the cooling curves seen in Fig. 7c reveal T_B_ beyond 400 K for the mCNTs, the interaction between ZnFe_2_O_4_ nanoparticles and Ni residuals in mCNTs is evident and causes T_B_ to reduce to 270 K. The broadening of T_B_ peak at 270 K is attributed to the aforementioned interaction rather than the well-known influence of particle size distribution [33]. The hysteresis loop of ZnFe_2_O_4_/mCNTs in Fig. 7d shows an increase in ferromagnetic behavior with coercivity of 501 Oe and saturation magnetization of nearly 2.5 emu/g at 4 K compared to that found at 77 K. This increase is due to the ferromagnetic behavior since 4 K is below the T_B_ of the sub-systems.

## Conclusion

Zinc ferrite nanoparticles have been encapsulated in mCNTs using wet chemistry technique. The composite was characterized using XRD, HRTEM, XPS, UPS, VSM, SQUID, and Mossbauer spectroscopy. The internal walls of CNTs were observed to partly coat the encapsulated zinc ferrite nanoparticles. The observed Ni impurities in the mCNTs are attracted to the zinc ferrite nanoparticles. This attraction provides a possibility of using encapsulated zinc ferrite nanoparticles to purify CNTs prepared using magnetic catalyst. In addition, Ni impurities are observed to be corresponding to the changes in electronic and magnetic properties. Decrease in the DOS of the ZnFe_2_O_4_/mCNTs indicates charge transfer from Ni impurities in the mCNTs to zinc ferrite nanoparticles in addition to the 0.3 eV shift of C1s core level. The ZnFe_2_O_4_/mCNTs composite exhibits two blocking temperatures and enhancement of magnetic properties, i.e., coercivity and magnetization remanence; resulting from its interacted ZnFe_2_O_4_ and Ni/mCNTs components.
